# Characterization of the VOC Promoter That Is Active Under Low-Salinity Conditions in the Diatom *Phaeodactylum tricornutum*

**DOI:** 10.3390/md23050185

**Published:** 2025-04-26

**Authors:** Charlotte Toustou, Carole Plasson, Marie-Christine Kiefer-Meyer, Muriel Bardor

**Affiliations:** 1 Laboratoire Glycobiologie et Matrice Extracellulaire Végétale (GlycoMEV) UR4358, University of Rouen Normandie (UNIROUEN), Normandie Université, 76821 Mont-Saint-Aignan, France; charlotte.toustou1@univ-rouen.fr (C.T.); carole.plasson@univ-rouen.fr (C.P.); 2 ALGA BIOLOGICS, Centre Universitaire de Recherche et d’Innovation en Biologie (CURIB), 25 Rue Tesnière, 76821 Mont-Saint-Aignan, France

**Keywords:** *Phaeodactylum tricornutum*, microalgae, promoters, episomal expression, synthetic biology, recombinant proteins, bioproduction

## Abstract

Microalgae such as *Phaeodactylum tricornutum* are promising cell biofactories for the production of high-value molecules, including monoclonal antibodies (mAbs). However, to date, the production of mAbs in *P. tricornutum* using the inducible nitrate reductase (NR) promoter has yielded only a limited amount of mAbs. Therefore, the identification of a robust promoter that produces high yields of mAbs is crucial for the development of a cost-effective expression system. To date, only a few endogenous promoters have been characterized in *P. tricornutum*. In this study, we identified thirty-three potential “strong” endogenous promoters based on our previously published transcriptomic data from the *P. tricornutum* Pt3 strain. These putative promoter sequences were cloned into an episomal vector and fused to the gene encoding enhanced green fluorescent protein (eGFP). Their strength was assessed by measuring eGFP fluorescence, which reflects the level of eGFP protein expression. Of the thirty-three promoters, thirteen were able to successfully drive eGFP protein expression. Among them, the best results were obtained with the VOC promoter, which allowed a significant increase in eGFP expression compared to that induced by the NR promoter. These results contribute to the identification of new genetic tools that can be used in future studies to increase the yield of production of recombinant proteins in *P. tricornutum* at an industrial scale.

## 1. Introduction

Diatoms are unicellular eukaryotic cells that are responsible for about 20% of the annual carbon dioxide fixation [1]. Among them, the marine diatom *P. tricornutum* is widely used as a study model for genetic engineering [2]. This microalgae is thought to have evolved through a series of secondary endosymbiotic events in which a cyanobacteria and then a red alga were engulfed by a heterotrophic eukaryote [2,3], thereby generating specific genetic and epigenetic traits [4,5,6]. Metabolic pathways belonging to either the plant or animal kingdom have also been described in *P. tricornutum* [7]. This allows biological traits that are responsible for the great adaptability of *P. tricornutum* [2,4,8] and its ability to grow in different culture conditions such as hyposaline conditions, low temperatures, or low light [9,10,11]. In addition, *P. tricornutum* has the property of being pleiomorphic, exhibiting three main morphotypes: fusiform, triradiate, and oval [9,10,12,13,14,15]. The fusiform morphotype is the main morphotype found in nature and is also the easiest one to maintain under laboratory culture conditions [10]. However, among the ten *P. tricornutum* strains whose genomes have recently been sequenced [4,5], some of them preferentially display specific morphotypes. For example, the Pt8 strain preferentially displays triradiate morphotype, whereas Pt3 and Pt9 strains most commonly display oval morphotype [10].

Several studies have highlighted the ability of *P. tricornutum* to act as a cost-effective cell biofactory [7] for the production of high value-added molecules, such as bioplastics [16], terpenoids [17,18,19,20], or biologics including monoclonal antibodies (mAbs) [21,22,23,24]. However, the development of the industrial-scale production of these molecules remains limited and requires an increase in production yield [18,20,21,23,24]. For example, the yield obtained for the production of a mAb directed against Hepatitis B antigen in *P. tricornutum* is lower than that obtained in Chinese Hamster Ovary (CHO) cells, which are currently the predominant host cells for mAbs production [23,25]. In addition, the simultaneous expression of multiple exogenous genes, such as the co-expression of both heavy and light chains that are necessary for mAb production, could be improved by using multiple promoters to express different proteins. In fact, using the same promoter to express multiple proteins could lead to transcriptional silencing [26,27]. In order to improve its production capacity, many studies are currently focused on the discovery and characterization of strong promoters in the *P. tricornutum* diatom, by assessing the fluorescence or activity of reporter proteins [28,29,30,31,32,33,34,35,36,37,38,39,40,41,42,43,44,45,46]. Classically, the characterization of these promoters was mainly performed with biolistic transformation, which induces random and variable copy number integration. In contrast, recent studies have characterized new promoters using the bacterial conjugation transformation, a method that allows episomal expression [28,29,45,46]. In fact, the use of episomal expression has been described as more robust and consistent for the study of transgene expression between transformants [20,45], although it allows lower transgene expression compared to the conventional biolistic method [20]. This is due to the presence of a single copy of the plasmid in the diatom cell and the non-integration of the foreign DNA into the genome, thus avoiding the transgene position effect. This method is advantageous for more reliable, controllable, and consistent genetic studies [20,47,48].

Thanks to the availability of data resulting from the genome sequencing of different strains of *P. tricornutum* [4,5], the identification of transcription factors [49,50,51,52,53], and the generation of extensive transcriptomic [5,13,54,55,56,57,58,59,60,61] and proteomic [62,63,64,65,66,67,68,69] data, more than twenty endogenous promoters have recently been characterized in *P. tricornutum,* in addition to the commonly used fcpA/B promoters [38,39,70,71] and the inducible nitrate reductase (NR) promoter [21,22,23,31]. Moreover, recent studies have taken advantage of transcriptomic datasets to identify new promoters leading highly expressed genes that can be used as tools in order to improve recombinant protein production in *P. tricornutum* [45,72].

In addition, recent studies have focused on the comparative study of the three morphotypes of the *P. tricornutum* Pt3 strain [13,14,69]. Among these morphotypes, the oval morphotype is cultivated under culture conditions where the salinity is ten times lower than that of the fusiform or triradiate morphotypes. This could be advantageous for the recombinant proteins secreted into the culture media. Indeed, the molarity of NaCl in the culture medium of fusiform or triradiate cells of *P. tricornutum* is almost four times higher than that observed in 1M phosphate-buffered saline (PBS), which is known to increase protein stability [73]. Thus, under these high-salt conditions, protein stability may be compromised, leading to protein precipitation or aggregation, which may challenge the efficiency of purification methods and limit the overall final production yield. In addition, the oval morphotype has been shown to be able to secrete proteins faster [14] and in larger amounts than the fusiform morphotype of the same strain [14,74]. Therefore, it has recently been suggested by Galas and co-workers that the oval morphotype may be a good candidate for the accumulation of recombinant proteins [14]. Although the presence of this oval morphotype seems to correlate with non-optimal culture conditions [10], it has been shown that Pt3 cultures enriched for the three morphotypes have similar growth curves [13]. Therefore, the identification of promoters that allow the expression of proteins of interest in a morphotype described as a better protein producer at a lower salt concentration could be of interest. In the present work, based on previous transcriptomic data comparing the three main morphotypes (fusiform, oval, and triradiate) of the Pt3 strain of *P. tricornutum* [13], we identified and characterized new *P. tricornutum* promoter sequences. The results lead to (1) increasing the repertoire of genetic elements available to the scientific community and (2) identifying promoters that can improve the production yields of recombinant proteins under low-salinity conditions.

## 2. Results and Discussion

### 2.1. Identification and Cloning of Potential Promoter Sequences

To identify potential strong promoter sequences expressed in the oval morphotype of *P. tricornutum* cultured in 10% seawater supplemented with Conway, we re-analyzed a previous RNA-seq study performed on the three morphotypes of the Pt3 strain of *P. tricornutum* [13]. Differentially expressed genes with a Log2FoldChange greater than five in a pairwise comparison of the oval morphotype with the fusiform and triradiate morphotypes were considered as highly overexpressed and were selected, resulting in fifty-five highly overexpressed genes (Supplementary Table S1). In silico analyses of these fifty-five genes were performed using the IGV [75] and Blast2GO [76] software to identify potential promoter sequences upstream of the overexpressed genes. The IGV software was used to visualize the alignment between transcript expression of the genes identified as overexpressed and *P. tricornutum* reference genome annotation (genome assembly ASM15095v2 and gene annotation Phaeodactylum_tricornutum.ASM15095v2.25.gff3 and Phaeodactylum_tricornutum.ASM15095v2.21.gff3). Genes for which the predicted coding sequences did not match the expression profiles of the transcripts were considered to be mispredicted. An example of what we consider to be a well-predicted or mispredicted coding sequence is shown in Supplementary Figure S1. In total, 22 sequences were mispredicted and were not conserved for the follow-up work presented in this study. In parallel, functional annotations were assigned to the fifty-five genes using the Blast2GO software. Eight out of the fifty-five genes (14.5%) had no functional annotation (Supplementary Table S1). These 8 genes were also among the mispredicted genes in the transcript expression analysis mentioned above. Therefore, they were excluded from the study. Altogether, these in silico analyses reduced the number of potential promoters to be analyzed to thirty-three (Table 1). For clarity, gene promoters for which a functional annotation could be determined were renamed according to this functional annotation. For genes for which only a predicted protein annotation was found, the gene name was retained and corresponds to the gene ID (Table 1).

Several promoter characterization studies in *P. tricornutum* have resulted in the selection of an approximately 500 bp region upstream of endogenous genes capable of driving the transgene expression [29,33,43]. However, it has also been shown in *P. tricornutum* that selecting a larger promoter region upstream of the gene allows for better transgene expression [32,34,44]. As a consequence, in this study, we considered the entire intergenic region between the STOP codon of the upstream gene and the ATG of the gene of interest as a putative promoter. The predicted lengths of these putative promoter sequences are given in (Table 1). The sequences of the thirty-three putative promoters were obtained from the ASM15095v2 version of the *P. tricornutum* CCAP 1055/1 genome available on the EnsemblProtists website (https://protists.ensembl.org/index.html, accessed on 11 September 2018). PCR amplification of these putative promoters was only possible for twenty-eight of them (Table 1). Moreover, for some of them, the control sequencing step revealed a small difference in length between the theoretical and the experimentally amplified sequence, as observed for some promoters such as P_SCF34_, P_SSR_, P_46468_, P_41599_, P_PCNTH_, and P_HDC_, for which differences of more than 10 nucleotides were observed between theoretical and experimentally amplified sequence (Table 1). For the SSR promoter, two bands were amplified: the expected band migrating at 1890 bp and a band at 1246 bp, representing a smaller size than expected. The purification yield of the expected size amplicon was insufficient to proceed further, so the 1246 bp amplicon was retained as a truncated version of the p37038 promoter to further evaluate if this shorter sequence could drive reporter gene expression. Thus, the 28 potential promoter sequences that could be amplified and validated by sequencing had an average length of 912 bp (Table 1), which is very close to the average size of *P. tricornutum* intergenic regions, which have been described to be between 1000 and 1500 bp [77].

### 2.2. Assessment of the Ability of the Potential Promoter Sequences to Drive eGFP Expression

To create a versatile vector that would allow a fair comparison of the 28 potential promoters, a plasmid vector compatible with the sequence and ligation-independent cloning (SLIC) method [78,79,80] was prepared. For this purpose, the pPtpuC3 transformation vector used for episome expression in *P. tricornutum* [47] was modified to introduce a ‘SLIC-eGFP-NRt’ transcription unit (TU) (Supplementary Figure S2). This TU contains the eGFP coding sequence, the NR terminator and a “SLIC” cassette, allowing easy cloning of promoters. The aim of our study was to compare the efficacy of new promoters in comparison to the NR promoter, while introducing as little bias as possible. We chose to retain the NR terminator, which has previously been used in conjunction with the NR promoter for mAb production in *P. tricornutum* [21,22,23]. The twenty-eight cloned potential promoters, validated by sequencing, were then amplified from the cloning vector with PCR using specific primers for subsequent cloning into the SLIC vector. The NR promoter, previously used for the production of mAbs in *P. tricornutum* [21,22,23], was also cloned into the SLIC vector and used as a reference to select promoters with higher strength. For all constructs, *P. tricornutum* colonies appeared on a selective medium between 10 and 15 days after transformation with bacterial conjugation. After two subcultures on a fresh agar medium with selective antibiotics, twenty transformants were screened with PCR using primers specific for the eGFP reporter gene. Sixteen positive PCR clones were used to assess the ability of the promoter to express eGFP. However, for cell lines receiving vectors containing the P_MI_ and P_DUF11_ promoters, only 12 and 7 clones, respectively, were used due to a lower number of colonies grown on the selective medium. Since the promoters tested in this study were isolated from genes overexpressed under low-salinity conditions, cells were grown in hyposaline medium (10% seawater) from colonies isolated on agar plates. To induce the NR promoter under comparable conditions, the cell line receiving the plasmid containing this reference promoter was placed in the same hyposaline medium containing NaNO_3_ to activate this nitrate-inducible promoter.

**Figure 1:**
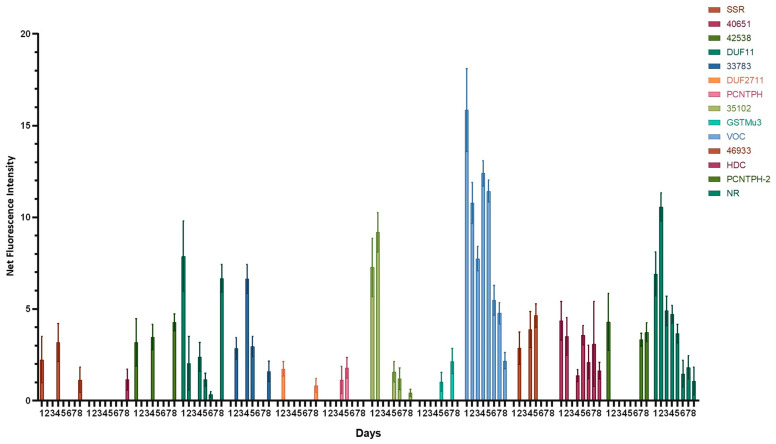
Levels of eGFP expression driven by novel promoters in *P. tricornutum* cells grown under low-salinity conditions (10% sea water). The results are presented as the mean of net eGFP fluorescence per OD_680nm_ unit in transformed *P. tricornutum* cell lines grown over 8 days under low-salinity conditions. Only promoters for which eGFP expression could be detected are shown.

Although conjugation is supposed to limit the variability of gene expression between clones, differences in the fluorescence level of a reporter protein of up to 30-fold between two independent clones from the same conjugation event have been reported [28]. For a fair assessment of promoter strength, up to 16 PCR-positive clones receiving the same construct were pooled and grown together for fluorometric analysis. A promoter-less vector (pPtPUc3_SLIC-eGFP-NRt) was used to ensure that the observed fluorescence was specifically due to eGFP expression driven by one of the tested promoters and not due to residual background from chlorophyll. For this purpose, the fluorescence emitted by the pool of cell lines transformed with the promoter-less vector was subtracted from the fluorescence emitted by each cell line pool. The mean net fluorescence of each transformed cell line pool is presented as normalized net fluorescence per OD_680nm_ unit (Figure 1).

To determine the profile of promoter efficiency in driving eGFP expression, the fluorescence intensity of each cell line pool was measured by fluorometry over a period of 8 days. As shown in Figure 1, the expression of eGFP driven by the NR promoter, our experimental reference, increased until it reached its maximum on day 2 after culturing the diatom cells in the presence of nitrate. This step was followed by a decrease in eGFP fluorescence from day 3 onwards. These results are consistent with previous findings observed in mAb production [23]. Interestingly, this result demonstrated that NR promoter presents the same activity profile regardless of the salinity of the medium used. The eGFP expression then continued to decrease until it stabilized at its lower level in the stationary phase of the culture.

Regarding the 28 putative promoter sequences tested, approximately half did not allow eGFP expression under the conditions tested in this study. This lack of expression may be due to the fact that the cultures were not maintained under low salinity conditions for a long period of time, unlike the culture used in the RNA-seq analyses of the three morphotypes [13]. Indeed, the initial hypothesis of our work was that only switching from a reference medium (100% seawater) to a hyposaline medium (10% seawater) would allow eGFP expression, without enriching the culture of *P. tricornutum* with oval cells. This working hypothesis is supported by another recent study suggesting that the difference in expression of some genes between oval morphotype and the other two Pt3 morphotypes could be mainly due to the hyposalinity of the medium, rather than to the morphotype itself [81]. For the remaining 13 sequences that allowed eGFP expression, different expression profiles were obtained. First, some promoters were only able to express eGFP after one day, and at a low level of expression. This is the case for promoters: P_40651_, P_DUF2711_ and P_PCNTH_, for which the fluorescence intensity is low and only detectable at day 8, day 2, and day 5, respectively. The P_SSR_, P_42538_, P_DUF11_, P_33783_, P_35102_, P_46933_, P_HDC_ and P_PCNTH-2_ promoters allowed a more cyclic and variable expression of eGFP but mostly with an intensity lower than the eGFP fluorescence obtained under the control of NR promoter. Regarding the activity of SSR promoter, no definitive conclusion can be drawn about its effectiveness. In fact, as mentioned above, we were only able to amplify and clone a 1246 bp version of the SSR promoter by PCR, which is 644 bp shorter than the theoretical version. Therefore, it is possible that some important regulatory elements were lost during the amplification of this promoter sequence. Thus, the band around 1,890 bp would need to be amplified in larger quantities to draw definitive conclusion about the P_SSR_. Finally, the VOC promoter seemed to stand out from the others and induced higher eGFP expression compared to the NR promoter. Therefore, we concluded that the VOC promoter had better activity than NR (Figure 1) in the culture conditions used in this study. However, it would be interesting to test these promoters in other conditions of culture and in conditions closer to the ones used in bioproduction at industrial scale, in particular in much larger culture volumes, to see if the expression profiles remain the same. Moreover, the stability and robustness of these promoters should be tested in future work.

### 2.3. Characterization of the VOC Promoter

#### 2.3.1. The Activity of the VOC Promoter Correlates with the Growth Phase of the Culture

**Figure 2:**
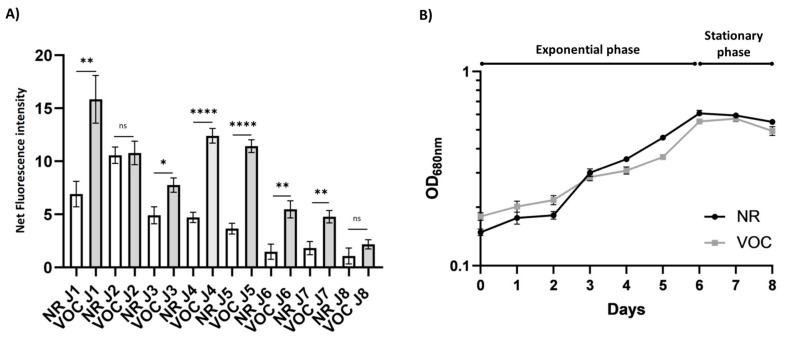
Comparison of the VOC and NR promoter activities during the growth phases. (**A**) Comparison of eGFP expression driven by the VOC and NR promoters over an 8-day culture period in low-salinity conditions (10% sea water). (**B**) Growth curves over an 8-day period of cultures containing pooled clones expressing eGFP either under the VOC or NR promoter. Error bars represent the standard error of means (SEM, *n* = 9 for every day except on day 5 where *n* = 8). Statistical differences were analyzed with a Welsh’s t-test comparison, a Mann–Whitney test for data following a normal distribution, or a Shapiro-Walk test for those not following a normal distribution, respectively. ns: *p*-value > 0.05, * *p*-value < 0.05, **: *p*-value < 0.01, ****: *p*-value < 0.001.

To further compare the activity of the VOC promoter with that of NR over time, the net eGFP fluorescence expressed by the VOC promoter was compared daily with that of NR. The NR promoter appeared to have maximum activity during the first two days of culture, with maximum activity on day 2. From day 3, eGFP expression decreased until it stabilized in the stationary phase from day 6 (Figure 2A,B). It seems that the activity of the NR promoter correlates with the presence of nitrate in the culture medium, regardless of the growth phase of the culture. Indeed, the maximum expression of proteins whose gene is under the control of this promoter always seems to occur in the first few days after its induction by the presence of NO_3_^-^ ions as nitrogen source, regardless of the day of culture [23,31,82]. A study conducted to evaluate eGFP expression by the NR promoter, showed that eGFP expression had the highest rate of increase within 3 to 6 h after transferring the culture into a medium containing NaNO_3_ as a nitrogen source [31].

In contrast, the expression of eGFP under the control of the VOC promoter correlates with the different phases of the growth curve (Figure 2A,B). Indeed, the VOC promoter seems to be active as soon as the cells are cultured under the low-salinity conditions and throughout the exponential growth phase. After maximum expression on the first day of culture, the fluorescence intensity decreased slightly and remained at the same average intensity until day 5. From day 6 onwards, eGFP expression decreased and reached the same intensity as that obtained with the NR promoter on the last day of culture. This time corresponds to the beginning of the stationary phase. According to the DiatOmicBase (https://www.diatomicsbase.bio.ens.psl.eu/, accessed on 6 November 2023 ), the Phatr3_J34085 gene, corresponding to the VOC gene (Table 1), is strongly under-expressed (Log2FoldChange: −4.55) in the stationary phase compared to the early growth phase of a Pt4 strain culture of *P. tricornutum*. Interestingly, according to the same database, nitrate and phosphate starvation or dark exposure does not seem to affect the expression of Phatr3_J34085, but light intensity can increase its expression. Moreover, by retrieving transcriptomic data from Truong and coworkers [83], in which the light intensity was increased from 20 µmol photons m^−2^ s^−1^ to 200 µmol photons m^−2^ s^−1^, it appears that the expression of this gene increases significantly. Considering the overexpression of the Phatr3_J34085 gene at higher light intensities, it could be interesting to optimize the activation of its promoter by increasing light intensity in further studies, since our study was performed at 30 µmol photons m^−2^ s^−1^. It has also been shown that genes encoding proteins of the VOC family are highly expressed under abiotic stress in plants such as *Arabidopsis thaliana* or rice [84]. Therefore, it could be interesting to test other forms of stress on the transformed *P. tricornutum* cell line to evaluate whether an increase in recombinant protein production is observed in response to stresses.

In addition, some studies have suggested that transgene expression may also depend on the terminator sequence used. Indeed, the effect of a promoter may differ depending on whether its native terminator or another terminator is used [29,45]. In this work, all the different promoters were compared using the NR terminator to avoid any bias in the analysis. In future studies, it would be interesting to compare the efficiency of the VOC promoter and its native terminator with the VOC promoter/NR terminator pair or another terminator, such as the fcpA one, and evaluate whether this could help improve the efficiency of the VOC promoter.

#### 2.3.2. In Silico Analyses of the VOC Promoter

**Figure 3:**
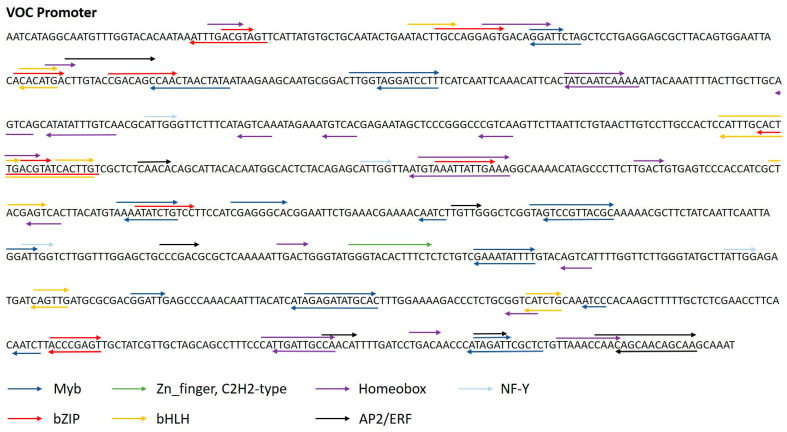
Transcription factor binding sites identified in the sequence of the VOC promoter of *P. tricornutum*. The analysis was performed using PlantPAN4.0 software. Results were analyzed also based on the transcription factors previously identified in *P. tricornutum* by [49], and [52]. Only sequences with a similarity score > 0.9 are shown.

Transcription factors (TFs) have been shown to be key elements in the regulation of gene expression in eukaryotes. Approximately 2% of the *P. tricornutum* proteome corresponds to TFs [49,52]. Therefore, it is important to better understand these elements in order to better control gene expression in *P. tricornutum*. Thus, an in silico analysis was performed to identify potential cis-acting regulatory elements present in the VOC promoter. To date, there is no database for identifying TF-binding sites on *P. tricornutum* promoters. Therefore, the PlantPAN 4.0 tool [85] was used to identify TF-binding sites according to the TF families previously identified in *P. tricornutum* [49,52]. Numerous binding sites were identified on the VOC promoter for four of the five most reported TFs in *P. tricornutum* (Figure 3), such as Myb, bZIP (basic leucine zipper), zinc-finger (C2H2 type), and bHLH (basic helix–loop–helix) binding sites, which represent on average 17.9%, 11.8%, 5.7%, and 3.7%, of the TFs identified in *P. tricornutum,* respectively [49,52]. Surprisingly, no binding sites for heat shock factors (HSFs) were identified (Figure 3), although they represent a major TF family (30–35%) in *P. tricornutum* [49,52]. TFs associated with the binding sites identified on the VOC promoter have been identified as being involved in stress responses in higher plants or in microalgae. Among them, bZIP TFs are conserved and present in several species. The role of bZIP TFs in stress response, such as light response [85,86] or salt stress [87,88,89], has been demonstrated in plants and microalgae. Some studies performed on *P. tricornutum* have also highlighted their role in gene expression during the light cycle, CO_2_ availability, or nitrogen starvation [35,48,90,91]. MYB proteins have been identified to be involved in cellular morphogenesis or in response to abiotic stresses, such as salinity or low temperature in plants [86]. Recently, a study carried out on *P. tricornutum* highlighted the presence of 26 PtMYB proteins. Some of them seem to be involved in adaptation to nitrogen deficiency and the light–dark cycle [87]. bHLH TFs have also been identified in the response to salt stress in plants [88,89]. It would be interesting to further investigate the involvement of TFs in the cellular response to salt stress. The generation of cell lines mutated in the target genes encoding TFs whose binding sites have been identified in the VOC promoter could thus help to definitively elucidate their functions.

## 3. Materials and Methods

### 3.1. Cell Culture and Growth Conditions

The *P. tricornutum* strain Pt3 (CCAP 1052/1B) was used for this study. Cells were grown at 19 °C under a 16 h/8 h light/dark cycle with a light intensity of 30 µmol photons m^−2^ s^−1^. Positive transformants were maintained on 1.5% agar plates containing 100% artificial seawater (33.3 g L^−1^ of sea salt (Instant Ocean^®^, Aquarius System, Sarrebourg, France)) filtered through 0.45 µm filters and sterilized using an autoclave. The sterilized culture medium was supplemented with 1 mL L^−1^ of Conway’s medium (Na_2_EDTA.2H_2_O: 45 g L^−1^; NaNO_3_: 100 g L^−1^; H_3_BO_3_: 33.6 g L^−1^; NaH_2_PO_4_: 20 g L^−1^; FeCl_3_: 0.768 g L^−1^; ZnCl_2_: 21 mg L^−1^; CoCl_2_, 6H_2_O: 20 mg L^−1^; (NH_4_)_6_Mo_7_O_24_, 4H_2_O: 9 mg L^−1^; CuSO_4_, 5H_2_O: 20 mg L^−1^; MnCl_2_, 4H_2_O: 360 mg L^−1^; B1 vitamin: 200 mg L^−1^; B12 vitamin: 10 mg L^−1^) and 80 mg L^−1^ of sodium metasilicate. In this manuscript, we named this medium “SAS medium” for Supplemented Artificial Seawater containing Conway medium and sodium metasilicate.

Expression of eGFP was performed on 5 mL cultures in 6-well plates containing 10% artificial seawater supplemented with the same proportions of Conway’s medium and sodium metasilicate. Three independent cultures for each promoter to be tested were grown at 19 °C with continuous shaking at 150 rpm on a 16 h/8 h light/dark cycle with a light intensity of 30 µmol photons m^−2^ s^−1^.

### 3.2. Putative Regulatory Sequences Selection

To identify putative promoter sequences, we used the results of a previous RNA-seq transcriptomic analysis of the three main morphotypes of the *P. tricornutum* Pt3 strain [13]. A selection of the most overexpressed genes (Log2 Fold Change values > 5) in the oval morphotype compared to the other two morphotypes was performed. In silico analyses of these genes and their upstream regulatory sequences were performed using the Integrative Genomics Viewer (IGV v2.11.4, Broad Institute, Cambridge, MA, USA) [75] and Blast2GO (v5.1, BioBam, Valence, Spain) [76] software to retain only sequences with a higher probability of being promoter regions. Transcriptomic data were visualized using IGV to identify and eliminate mispredicted sequences. The Blast2GO software was used to verify the sequence description, and sequences that could not be annotated, even as “predicted proteins”, were also removed from the study.

### 3.3. Isolation of Putative Promoter Regions

DNA extraction from the Pt3 strain was performed using the Nucleospin Plant II kit (Macherey-Nagel, Düren, Germany), PL1 lysis buffer was used, and the cells were lysed using lysing beads (E-matrix lysing tubes, MP Biomedicals^®^, Fisher Scientific, Illkirch, France) and ground for 3 cycles of 30 s at 6.5 m s^−1^ in a FastPrep-24^TM^ homogenizer (MP Biomedicals^®,^ Fisher Scientific, Illkirch, France). The rest of the extraction was carried out according to the manufacturer’s instructions. Putative promoter sequences were amplified with PCR from genomic DNA using the Phusion Green Hot Start II High Fidelity DNA Polymerase (ThermoFisher Scientific, Illkirch, France). Primers used for amplification were synthesized by Eurogentec (Kaneka Eurogentec S.A., Seraing, Belgium) and are listed with their hybridization temperature in Supplementary Table S2. Amplicons were purified using the “Wizard SV Gel and PCR Clean-Up System” (Promega, Charbonnières-Les-Bains, France) and cloned into either the pJET1.2/blunt cloning vector (ThermoFisher Scientific) or in the SmaI linearized pUC19 vector (ThermoFisher Scientific). Cloning into the pJET1.2/blunt cloning vector was performed according to the CloneJET PCR Cloning Kit instructions (ThermoFisher Scientific), and cloning into the pUC19 was performed using T4 DNA ligase (Promega). Cloning vectors were used to transform DH5α chemically competent *Escherichia coli* (Subcloning efficiency^TM^—Invitrogen, Illkirch, France). Clones were screened with colony PCR using the GoTaq G2 polymerase (Promega) and primers as described above. Plasmids were purified from *E. coli* cells using the Nucleospin Plasmid Kit (Macherey-Nagel). Finally, the putative promoter sequences were sequenced using the Sanger sequencing method prior to further experiments (Eurofins, Ebersberg, Germany).

### 3.4. Transformation Vectors Construction

The pPtpuC3 vector backbone [47] required for *P. tricornutum* transformation by conjugation was modified by insertion of a “SLIC-eGFP-NRt” transcription unit (TU) to clone the different promoters to be tested in this study. Based on previous work, the TU was amplified from a pNR-eGFP-NRt vector using the following primers: forward, 5′-GCT-CTA-GAT-AGT-TGG-AAT-GGT-ACG-TAC-CAA-CTC-CAT-AAG-GAT-CCA-TGG-TGA-GCA-AGG-GCG-AGG-AGC-3′, and reverse, 5′-ACA-TGC-ATG-CTC-CGG-ATG-CGT-TCA-TTT-TAG-ATC-CTG-ATC-CG-3′. Briefly, to construct the pNR-eGFP-NRt vector, the eGFP DNA sequence was recovered from the pPhaT1-eGFP vector [70] and was cloned into the pPha-NR vector (JN180663.1) optimized using e-Zyvec technology (Sartorius, Loos, France). After digestion of the pPtpUC3 vector backbone and the cassette “SLIC-eGFP-NRt” TU with the restriction enzymes SphI and XbaI, the cassette was ligated into the pPtPuc3 vector using T4 DNA polymerase (Promega), allowing the construction of the pPtPUc3_SLIC-eGFP-NRt vector (Supplementary Figure S2).

To construct the final vectors, the pPtPUc3_SLIC-eGFP-NRt vector was digested with the restriction enzyme SnaBI (Promega), and the putative promoter regions were amplified from the previously sequenced vectors (see Section 3.3.) using the primers listed in Supplementary Table S3. The putative upstream regulatory sequences were cloned into the pPtPUc3_SLIC-eGFP-NRt vector using the sequence and ligation-independent cloning (SLIC) method by adapting the method described previously [80]. The insert: vector ratio was increased to 10: 1 and 2 units of T4 DNA polymerase (Promega) were used. All the resulting SLIC vectors (empty or promoter-containing) constructed were also checked by Sanger sequencing (Eurofins, France) for validation prior to *P. tricornutum* transformation.

### 3.5. Transformation of P. tricornutum by Bacterial Conjugation

First, an *E. coli* strain containing the pTA-Mob (mobilization helper) vector was transformed with SLIC vectors (empty or containing the various promoters of interest). Transformation of *P. tricornutum* with bacterial conjugation was adapted from Karas and coworkers [47]. Exponentially growing cultures of transformed *E. coli* (37 °C in LB medium (Duchefa Biochemie, Haarlem, Netherlands)) and *P. tricornutum* (19 °C in SAS medium) were harvested using centrifugation (10 min at 3 000 g). Cell pellets were resuspended in 500 µL of SOC medium (Duchefa Biochemie) and 50% SAS medium for bacteria and diatoms, respectively. A total of 200 µL of both prokaryotic and eukaryotic cells were mixed and plated on 1% agar plates containing 50% SAS medium without silica and 5% LB. The agar plates were incubated at 30 °C for 1 h 30 min in the dark and then at 19 °C in a 16 h/8 h light/dark cycle for 72 h. Then, 1 mL of 50% SAS medium was added to the plates and the cells were scraped and resuspended. A total of 200 µL of resuspended cells were plated on selective agar plates containing 50% SAS medium and 20 µg ml^−1^ of phleomycin (Fisher BioReagents, Illkirch, France). Plates were incubated at 19 °C in a 16 h/8 h light/dark cycle, and colonies of transformed *P. tricornutum* appeared approximately 10 to 15 days after the transformation.

### 3.6. Confirmation of Transgene Presence by PCR Amplification

After transformation, colonies growing on selective medium were subcultured on fresh 1.5% agar plates containing SAS medium with 75 µg ml^−1^ of Zeocin. Prior to PCR screening, rapid DNA extraction was performed on approximately 20 Zeocin-resistant clones. For this purpose, a small amount of biomass from each individual clone was scraped directly from the agar plates and placed in 30 µL of lysis buffer (1% NP-40, 10 mM Tris-HCl, pH 8; 1 mM anhydrous EDTA; 1 M NaCl). Lysis was achieved by heating the cells at 95 °C for 10 min. Lysed cells were then vortexed briefly, and 120 µL of DNase-free water was added. DNA extracts were stored at 4 °C until further use. PCR amplification was performed using GoTaq^®^ G2 DNA Polymerase (Promega). Then, 1 µL of each DNA extraction was used and the manufacturer’s recommendations were adjusted to a final volume of 20 µL. The presence of the sequence encoding eGFP in the transformants was confirmed using the PCR primer pair 5′-ATCATGGCCGACAAGCAGAA-3′ and 5′-GACTGGGTGCTCAGGTAGTG-3′. Amplifications parameters were chosen according to the manufacturer’s guidelines. The selected hybridization temperature was 60 °C, and the elongation time was 45 s. Amplicons were analyzed on 1.5% agarose gels.

### 3.7. Assessment of the Activity of Potential Promoter Sequences by eGFP Fluorescence Intensity Measure

For the same construct, 16 independent clones were grown together in pools in 6-well plates containing 5 mL of culture, except for PMI and PDUF11, for which only 7 and 12 PCR-positive clones were obtained, respectively. In total, 150 µL of each culture was sampled daily to measure cell density (OD_680nm_) and eGFP fluorescence for 8 days. Measurements were performed in black, clear-bottomed 96-well plates (Costar^®^, Corning ^TMity^, Fischer Scientific, Illkirch, France). Transformants were screened through excitation of eGFP at 485 nm/20 nm, and emission was measured at 538/20 nm using a fluorometer (FlexStation 3 MultiMode Microplate Reader—Molecular Devices, San Jose, CA, USA). A cut-off filter of 530 nm was used. The eGFP fluorescence emitted under the control of each promoter was normalized to one OD unit (taking into account the OD_680nm_ linearity region). The normalized fluorescence of the empty vector control (pPtPUc3_SLIC-eGFP-NRt) was subtracted from each condition in order to ignore the chlorophyll background signal. All measurements were performed with three technical replicates on three independent biological replicates.

### 3.8. Statistical Analyses

Means of net eGFP fluorescence per OD_680nm_ unit, means of the growth curve, standard errors of the means (SEM), and statistical analyses were calculated and performed using GraphPad Prism software (v.8.0.2, Boston, MA, USA). After analyzing the expression of eGFP by the different promoters tested, statistical analyses were performed on the best promoter to compare it with the reference promoter chosen in our study, namely the NR promoter Therefore, the normalized net eGFP fluorescence intensity of the VOC promoter was compared with that of the reference NR promoter, over the 8 days culture period. The Shapiro–Wilk test was used to assess whether or not the data were normally distributed. The difference between the values obtained on different days for the NR and VOC promoters was evaluated using a Welsh’s *t*-test comparison for values following a normal distribution and using a Mann–Whitney test for values not following a normal distribution.

### 3.9. In Silico Analysis of Validated Promoter Sequences

Sequences that drove eGFP expression were further analyzed in silico. Prediction of cis-regulatory elements in these sequences was performed using the PlantCARE database [90]. Additional analysis was performed on promoters that allowed higher eGFP expression than that obtained with the NR promoter to characterize the binding sites of transcription factors (TFs) whose genes have already been identified in *P. tricornutum* [49] using PlantPAN2.0 (Plant Bioinformatics and Molecular Biology Laboratory, National Cheng Kung University, Tainan, Taiwan) [91].

## 4. Conclusions

*P. tricornutum* is now considered an emerging cell biofactory for the production of high value-added molecules including recombinant proteins [7]. However, the low production yield and the lack of strong promoters still represent a bottleneck that currently hinders industrialization. In addition, the high-salt concentration of *P. tricornutum* media could challenge further purification processes. In this study, we aimed to identify and characterize endogenous promoters in *P. tricornutum* that are active under low-salt conditions. By re-analyzing the transcriptomic data of the three morphotypes of Pt3 [13], we identified and tested 28 potential promoter sequences, 13 of which were capable of driving eGFP expression with different expression patterns. Among these, the VOC promoter showed significantly higher expression levels compared to NR, suggesting its potential as a strong candidate for recombinant protein production. These results enrich the genetic toolbox available for biotechnological applications in diatoms and offer a promising route to improve production yields under less saline conditions. Future research could explore the synergistic effects of VOC promoter–terminator combinations and test promoter performance under different abiotic stress conditions or light conditions, since many binding sites of TFs involved in stress response are present along the promoter sequence. This opens the door for further advances in algal biotechnology and synthetic biology.

## Figures and Tables

**Table 1:** Information regarding the 33 potential promoter sequences selected from a previous RNA-seq transcriptomic data analysis of the three morphotypes of the *P. tricornutum* Pt3 strain [13].

Gene ID	Protein Annotation (Blast2GO)	Promoter Name	Chromosomal Location	Promoter Predicted Length ^1^	Promoter Final Length ^2^
Phatr3_J39391	predicted protein	P_39391_	19:379218–380555	896 bp	896 bp
Phatr3_J33266	solute carrier family 34	P_SCF34_	3:462819–464704	1741 bp	1701 bp
Phatr3_J37038	syringomycin synthesis regulator	P_SSR_	12:20667–21773	1890 bp	1246 bp
Phatr3_J46468	predicted protein	P_46468_	10:427467–428684	817 bp	828 bp
Phatr3_Jdraft1668	taurine catabolism family	P_TC_	bd_31x35:110713–112101	560 bp	559 bp
Phatr3_J40651	predicted protein	P_40651_	25:73019–73327	756 bp	756 bp
Phatr3_J42538	major intrinsic	P_MI_	1:361483–362842	1089 bp	1092 bp
Phatr3_J48356	DUF11 domain-containing	P_DUF11_	17:217814–220228	986 bp	985 bp
Phatr3_J33783	predicted protein	P_33783_	4:340826–341837	747 bp	744 bp
Phatr3_J41599	predicted protein	P_41599_	32:49730–50980	1087 bp	1076 bp
Phatr3_J36794	predicted protein	P_36794_	11:381859–382908	1491 bp	^3^
Phatr3_J50361	predicted protein	P_50361_	29:82531–83523	755 bp	754 bp
Phatr3_J43621	DUF2711 family	P_DUF2711_	2:1005210–1005785	478 bp	477 bp
Phatr3_J34976	alkaline phosphatase	P_AP1_	6:788784–789464	681 bp	681 bp
Phatr3_J43494	predicted protein	P_43494_	2:626125–627632	1669 bp	^3^
Phatr3_J47869	alkaline phosphatase	P_AP2_	15:296103–298532	1972 bp	^3^
Phatr3_EG02507	RING-H2 finger ATL74-like	P_RHFA_	14:535986–536588	830 bp	830 bp
Phatr3_J8683	peptide methionine sulfoxide reductase	P_PMSR_	1:241098–242192	597 bp	596 bp
Phatr3_Jdraft1443	P-loop containing nucleoside triphosphate hydrolase	P_PCNTH_	bd_32x35:212637–213710	1138 bp	1125 bp
Phatr3_J40433	solute carrier family 34	P_SCF34-2_	23:381101–383068	735 bp	727 bp
Phatr3_J35771	predicted protein	P_35771_	8:716893–717834	1215 bp	^3^
Phatr3_J35102	predicted protein	P_35102_	7:63434–63919	535 bp	535 bp
Phatr3_J8537	type VI secretion system tip	P_SST5_	5:930561–935372	1542 bp	1542 bp
Phatr3_J36570	serine hydrolase	P_SH_	10:784031–785449	717 bp	725 bp
Phatr3_J50252	glutathione S-transferase Mu 3	P_GSTMu3_	28:107611–109050	1095 bp	1098 bp
Phatr3_J49693	glycerophosphodiester phosphodiesterase	P_GPPD_	23:448289–450075	860 bp	^3^
Phatr3_J34085	Vicinal Oxygen Chelate family	P_VOC_	4:1061396–1062934	957 bp	957 bp
Phatr3_J40539	predicted protein	P_40539_	24:193701–194375	687 bp	687 bp
Phatr3_J46933	predicted protein	P_46933_	11:854712–855482	1767 bp	1772 bp
Phatr3_J36444	predicted protein	P_36444_	10:464796–465641	851 bp	851 bp
Phatr3_J48164	hemerythrin domain-containing	P_HDC_	16:393379–394224	905 bp	872 bp
Phatr3_EG01906	predicted protein	P_E1906_	26:436989–438052	479 bp	472 bp
Phatr3_EG02422	P-loop containing nucleoside triphosphate hydrolase	P_PCNTH-2_	bd_32x35:126016–129603	953 bp	953 bp

^1^ Theoretical lengths of upstream intergenic sequences of genes overexpressed in the oval morphotype grown under low-salinity conditions. ^2^ Experimental lengths of upstream intergenic sequences of genes overexpressed under low-salinity conditions, obtained after cloning and sequencing. ^3^ Potential promoters for which PCR amplification from genomic DNA was not obtained or for which the DNA sequencing results were inconclusive.

## Data Availability

The original data presented in the study are included in the article and in the supplementary material, further inquiries can be directed to the corresponding authors.

## Supplementary Materials

The following supporting information can be downloaded at: https://www.mdpi.com/article/10.3390/md23050185/s1, Figure S1: Examples of sequence prediction analyses against transcript expression profiles using IGV software (v2.11.4); Figure S2: Map of the pPtPUc3_SLIC-eGFP-NRt vector used for the eGFP expression under the control of the different promoters; Table S1: List of the 55 genes with a Log2 Fold change greater than 5 between the oval and the triradiate morphotype (OT) or the fusiform morphotype (OF) according to the datasets of Ovide et al., 2018 [13]; Table S2: List of primers used in this study to amplify the intergenic sequences upstream of the 33 genes of interest selected by in silico analyses retrieved from the datasets of Ovide et al., 2018 [13]; Table S3: List of primers used in this study to amplify the putative promoter region sequences and the NR promoter, our reference in this study, in order to clone them into the pPtPUc3_SLIC-eGFP-NRt vector.
