# Supplementary material for: Characterization of the VOC Promoter That Is Active Under Low-Salinity Conditions in the Diatom Phaeodactylum tricornutum
Source: Mar Drugs. 2025 Apr 26;23(5):185. doi: 10.3390/md23050185 (PMC12113381; doi:10.3390/md23050185)
Supplement: Supplementary file 1 [file marinedrugs-23-00185-s001.zip › marinedrugs-3538215-supplementary.pdf]

Supplementary data

Characterization of the VOC Promoter That Is Active Under Low-Salinity Conditions in the Diatom *Phaeodactylum tricornutum*

Charlotte Toustou [1], Carole Plasson [1], Marie-Christine Kiefer-Meyer [1,*] and Muriel Bardor [1,2,*]

Supplementary Table 1. List of the 55 genes with a Log2 Fold Change greater than 5 between the Oval and the Triradiate morphotype (OT) or the Fusiform morphotype (OF) according to the datasets of Ovide et al., 2018.

Gene ID (EnsemblProtists)	BLAST2GO Annotation	Log2FoldChange OT	Log2FoldChange OF
Phatr3_J39391|Phatr3_J39391.t1||B7G8I7_B1PJ50	predicted protein	11,46887093	9,342275376
Phatr3_J33266|Phatr3_J33266.t1||B5Y4T7	solute carrier family 34	10,69208198	11,07922629
Phatr3_EG00380|Phatr3_EG00380.t1	---NA---	9,309692597	7,714279443
Phatr3_J33569|Phatr3_J33569.t1||B5Y5N2	gag-pol poly	9,179361508	7,152162845
Phatr3_J37038|Phatr3_J37038.t1||B7G1Y9	syringomycin synthesis regulator	8,854838715	7,194181044
Phatr3_J46486|Phatr3_J46486.t1||B7G1E2	predicted protein	7,998019717	6,15917011
Phatr3_EG00958|Phatr3_EG00958.t1	hypothetical protein FisN_22Lu255	7,898793597	5,995350969
Phatr3_Jdraft1668|Phatr3_Jdraft1668.t1||B7S431	taurine catabolism family	7,890425876	5,671758547
Phatr3_J39390|Phatr3_J39390.t1||B7G8I6_B1PJ51	pol protein, partial	7,845420664	6,619243434
Phatr3_J40651|Phatr3_J40651.t1||B7GC18	predicted protein	7,57159213	6,49157558
Phatr3_J42538|Phatr3_J42538.t1||B7FRM9	major intrinsic	7,533926295	5,862701345
Phatr3_J45819|Phatr3_J45819.t1||B7FYS5	predicted protein	7,474422891	6,059117137
Phatr3_J48356|Phatr3_J48356.t1||B7G6V0	DUF11 domain-containing	7,452005692	8,627085492
Phatr3_J48943|Phatr3_J48943.t1||B7G8Y5	tetratricopeptide repeat 19 mitochondrial isoform X2	7,451939297	6,11232305
Phatr3_J33783|Phatr3_J33783.t1||B7FTV2	predicted protein	7,397721761	6,08324856
Phatr3_J41599|Phatr3_J41599.t1||B7GEM9	predicted protein	7,26468955	6,286458233
Phatr3_J36794|Phatr3_J36794.t1||B5Y3P5	predicted protein	7,251180238	5,270289023
Phatr3_J50361|Phatr3_J50361.t1||B7GDV9	predicted protein	7,245309726	6,242843471
Phatr3_J43621|Phatr3_J43621.t1||B7FSW5	DUF2711 family	7,084193857	6,4175397
Phatr3_J34976|Phatr3_J34976.t1||B7FX80	glutathione S-transferase Mu 3	7,010194406	5,473092354
Phatr3_J43494|Phatr3_J43494.t1||B7FSF0	predicted protein	6,926537783	5,420176761

Phatr3_EG01105|Phatr3_EG01105.t1	---NA---	6,879106824	7,301912884
Phatr3_J47869|Phatr3_J47869.t1||B7G571	alkaline phosphatase	6,828309618	6,109272934
Phatr3_EG02507|Phatr3_EG02507.t1||B7G4K2	RING-H2 finger ATL74-like	6,81150837	5,49365969
Phatr3_J8683|Phatr3_J8683.t1||B7FRI4	peptide methionine sulfoxide reductase	6,642507784	5,917676515
Phatr3_EG02634|Phatr3_EG02634.t1	predicted protein	6,640771947	6,110567161
Phatr3_J39571|Phatr3_J39571.t1||B7G912	predicted protein	6,502601408	5,261950224
Phatr3_J35939|Phatr3_J35939.t1||B7FZX8	SDR family oxidoreductase	6,46623113	5,134222296
Phatr3_Jdraft1443|Phatr3_Jdraft1443.t1||B7S4C2	P-loop containing nucleoside triphosphate hydrolase	6,441306607	5,708874825
Phatr3_EG01226|Phatr3_EG01226.t1	---NA---	6,359878785	5,321772373
Phatr3_J34433|Phatr3_J34433.t1||B7FW12	predicted protein	6,232356475	5,889188879
Phatr3_J40433|Phatr3_J40433.t1||B7GBF0	solute carrier family 34	6,143838119	8,250049817
Phatr3_J35771|Phatr3_J35771.t1||B7FZ77	predicted protein	6,109063984	6,438574936
Phatr3_EG00795|Phatr3_EG00795.t1	---NA---	6,079130624	5,854755874
Phatr3_J35102|Phatr3_J35102.t1||B7FXK3	predicted protein	6,033806198	6,344261425
Phatr3_J41393|Phatr3_J41393.t1	---NA---	6,010758094	5,593703745
Phatr3_J8537|Phatr3_J8537.t1||B7FV78	type VI secretion system tip	5,977305288	5,727219897
Phatr3_J36570|Phatr3_J36570.t1||B7G1Q6	serine hydrolase	5,968586624	5,752651162
Phatr3_J23830|Phatr3_J23830.t1	phosphate transporter	5,961141024	5,966914736
Phatr3_J50252|Phatr3_J50252.t1||B7GDG5	Aldo keto reductase	5,913700797	5,301146933
Phatr3_J49693|Phatr3_J49693.t1||B7GBH3	glycerophosphodiester phosphodiesterase	5,877471284	5,327816127
Phatr3_J34085|Phatr3_J34085.t1||B7FUP4	VOC family	5,815291624	5,924098031
Phatr3_J40539|Phatr3_J40539.t1||B7GBT5	predicted protein	5,748420027	5,922515176
Phatr3_EG02319|Phatr3_EG02319.t1||B7FXM7	predicted protein	5,730296993	5,41100342
Phatr3_Jdraft1747|Phatr3_Jdraft1747.t1||B7S4D7	ATP-grasp domain-containing	5,574035521	5,908619691
Phatr3_J46933|Phatr3_J46933.t1||B5Y492	predicted protein	5,507924375	5,775111399
Phatr3_EG02483|Phatr3_EG02483.t1||B7G8N3	predicted protein	5,501271474	5,088861976
Phatr3_J36444|Phatr3_J36444.t1||B7G1D2	predicted protein	5,481348892	5,247462135
Phatr3_J48164|Phatr3_J48164.t1||B7G675	hemerythrin domain-containing	5,412978068	5,642358478
Phatr3_EG00990|Phatr3_EG00990.t1	---NA---	5,295244115	5,237024442
Phatr3_EG00587|Phatr3_EG00587.t1	---NA---	5,271577107	5,115753876
Phatr3_J49862|Phatr3_J49862.t1||B7GC40	predicted protein	5,264902491	5,451983067
Phatr3_EG00533|Phatr3_EG00533.t1	---NA---	5,248113869	5,320152461
Phatr3_EG01906|Phatr3_EG01906.t1	predicted protein	5,247697144	5,091492335
Phatr3_EG02422|Phatr3_EG02422.t1||C6JVY5_B7S495	P-loop containing nucleoside triphosphate hydrolase	5,011803985	5,001125684

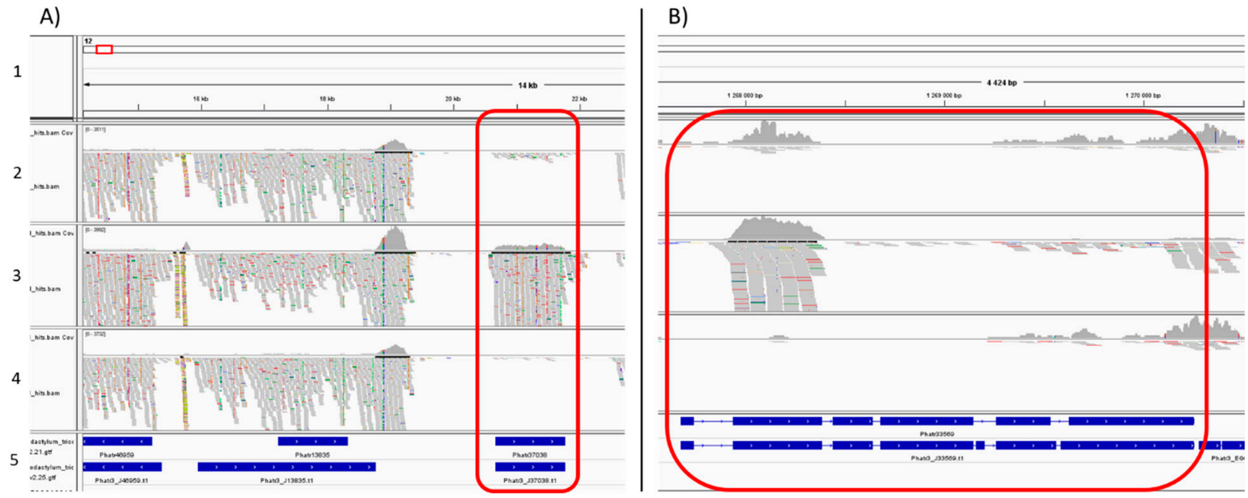

Supplementary Figure 1. Examples of sequence prediction analyses against transcript expression profiles using IGV software (v2.11.4). A) Example of a gene for which we considered that the coding sequence was well predicted, since it matches to the transcript expression profile. **B)** Example of a gene for which we considered that the coding sequence was mispredicted as it did not match the transcript expression profile.

<u>**Caption:**</u>

1 – Genome of *P. tricornutum* (genome assembly ASM15095v2);

2 – Transcripts expression profiles of the fusiform morphotypes retrieved from Ovide *et al.*, 2018;

3 – Transcripts expression profiles of the oval morphotypes retrieved from Ovide *et al.*, 2018;

4 – Transcripts expression profiles of the triradiate morphotypes retrieved from Ovide et al., 2018;

5 – Annotations of the *P. tricornutum*'s genome (Phaeodactylum_tricornutum.ASM15095v2.21.gff3 and Phaeodactylum_tricornutum.ASM15095v2.25.gff3, respectively).

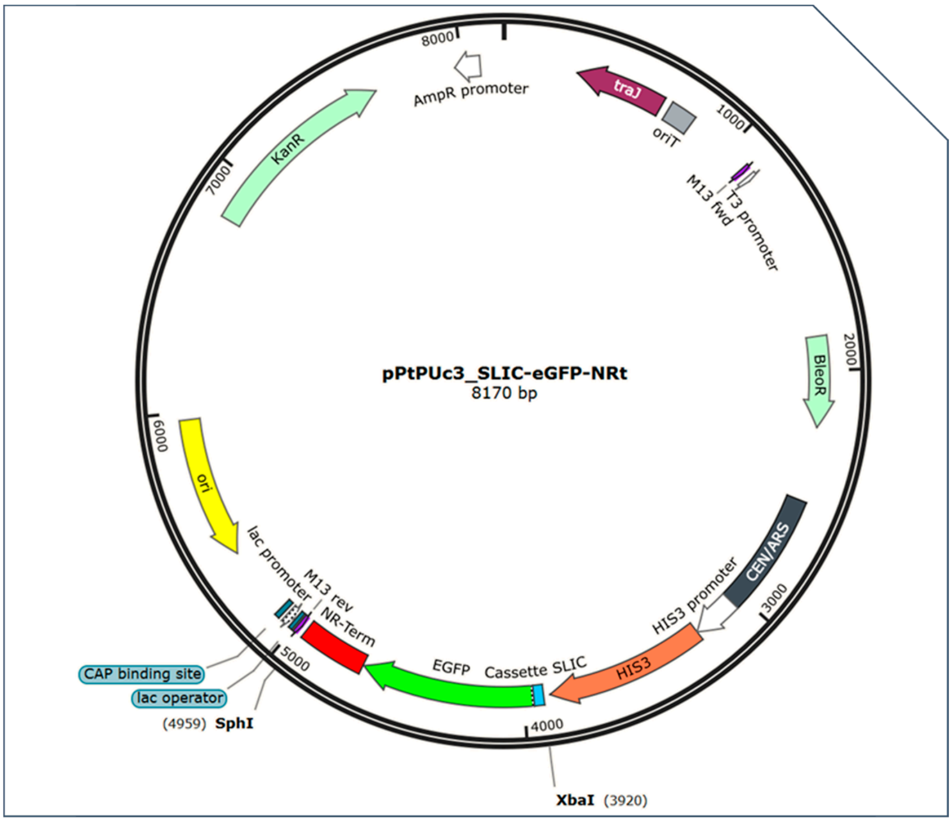

Supplementary Figure 2. Map of the pPtPUc3_SLIC-eGFP-NRt vector used for the eGFP expression under the control of the different promoters tested.

Antibiotic resistance cassettes are indicated in light green. Kanamycin resistance (KanR) was used to transform and select the *E. coli* strain containing the pTA-Mob vector. Zeocin Resistance (BleoR) was used to select transformants of *P. tricornutum* on selective medium. The episomal replication cassette is represented by the CEN/ARS-HIS3 sequence highlighted in dark grey. The SLIC cassette allowing the cloning of the promoters to be tested, is shown in cyan blue, the eGFP sequence in green and the NR terminator (NRt) in red.

Supplementary Table 2. List of primers used in this study to amplify the intergenic sequences upstream of the 33 genes of interest selected by *in silico* analyses retrieved from the datasets of Ovide *et al.*, 2018.

Target sequence	Primer's name	5'-3' nucleotide sequences	Hybridization temperature
Intergenic sequence upstream of the Phatr3_J39391 gene	For_J39391	AAT-GAT-AGT-TGG-TTA-GAT-TG	52°C
	Rev_J39391	TGG-AAT-TGG-ATT-AGA-TCT-GG	
Intergenic sequence upstream of the Phatr3_J33266 gene	For_J33266	TTT-GAT-TTG-GTT-CGT-CGT-CAA-C	48°C
	Rev_J33266	GGT-TCG-GAA-AAG-TCT-ACC-AGG-T	
Intergenic sequence upstream of the Phatr3_J37038 gene	For_J37038	TGT-CGC-TAC-TTC-ACC-ACA-GC	60°C
	Rev_J37038	GAT-TCA-GCC-TTG-AAT-CAC-GG	
	For_J46486	CTG-AAT-AGA-AAT-ATT-TCC-TCC-G	48°C

Intergenic sequence upstream of the Phatr3_J46468 gene	Rev_J46486	GGA-GGC-TTT-TGA-AGT-TTT-TTA-T	
Intergenic sequence upstream of the Phatr3_Jdraft1668 gene	For_Jdraft1668	CAT-TGT-CCT-ACA-TTG-ATG-AT	50°C
	Rev_Jdraft1668	AGC-AAT-GTA-AGT-AAT-TTG-AT	
Intergenic sequence upstream of the Phatr3_J40651 gene	For_J40651	ACT-TGC-GTT-CAT-CTC-AAG-TC	52°C
	Rev_J40651	AGT-GAT-ACA-ACA-AAA-TAG-CA	
Intergenic sequence upstream of the Phatr3_J42538 gene	For_J42538	GAA-GAT-GAG-CAG-TTT-CCG-CAT-C	50°C
	Rev_J42538	GGT-GGT-TGA-GAT-CCG-GGA-TCG-A	
Intergenic sequence upstream of the Phatr3_J48356 gene	For_J48356	CCT-TTA-GGA-GAA-CAA-GCT-GCG-A	50°C
	Rev_J48356	CTT-CGC-TGC-AGC-CTT-TTG-GTA-A	
Intergenic sequence upstream of the Phatr3_J33783 gene	For_J33783	GAC-CGT-GAC-GAC-GGA-TTG-TTT-T	48°C
	Rev_J33783	TGT-TGT-CGA-ATA-ATG-ATG-GCA-C	
Intergenic sequence upstream of the Phatr3_J41599 gene	For_J41599	GAT-GAG-TAC-AGA-TCA-GGC-ACA-A	48°C
	Rev_J41599	GAT-GTT-AAC-TAG-AAA-ACG-AAT-C	
Intergenic sequence upstream of the Phatr3_J36794 gene	For_36794	CGT-CAC-TTG-CTC-TTT-CTT-AAC-T	52°C
	Rev_36794	CGT-GAT-GCT-CGC-AGG-AAC-GAG-A	
Intergenic sequence upstream of the Phatr3_J50361 gene	For_J50361	CGT-CCT-TAC-GGC-CCC-TTT-GT	56°C
	Rev_J50361	GTC-AAT-AAA-CGA-TCT-CCT-GA	
Intergenic sequence upstream of the Phatr3_J43621 gene	For_J43621	ACC-ACG-TCA-ATT-GCG-TAA-TTC-G	48°C
	Rev_J43621	GGT-GCG-GAA-ATC-AAG-TTT-TCG-A	
Intergenic sequence upstream of the Phatr3_J34976 gene	For_J34976	TGA-CCA-AGG-TAC-GAA-GCT-CT	58°C
	Rev_J34976	TGG-TTT-GTC-ATC-AAC-TGG-CT	
Intergenic sequence upstream of the Phatr3_J43494 gene	For_J43494	GCC-GTA-TGA-TGC-GCT-TCA-TTC-A	48
	Rev_J43494	TTT-GAA-GCC-GAA-AAA-ACG-CGA-T	
Intergenic sequence upstream of the Phatr3_J47869 gene	For_J47869	TAA-GTT-TTC-TTA-CTG-TTG-AAA-A	Ramp from 48°C to 52°C
	Rev_J47869	CGA-AGA-ATT-CTT-TTC-ACC-AGG-G	
Intergenic sequence upstream of the Phatr3_EG02507 gene	For_EG02507	TTC-CGA-TGC-GTG-TGT-TTG-TAA-A	52°C
	Rev_EG02507	TTT-GTG-GTT-CGA-CGC-GAA-TCA-T	
Intergenic sequence upstream of the Phatr3_J8683 gene	For_J8683	GTT-GGG-AGA-ACC-ACT-ATC-GA	58°C
	Rev_J8683	GAG-ATT-TGT-GTG-GTC-ATA-CC	
	For_Jdraft1443	TCG-GGT-TGG-AGT-ACT-GCT-AG	60°C

Intergenic sequence upstream of the Phatr3_Jdraft1443 gene	Rev_Jdraft1443	AGT-CGT-GGA-ATG-GAT-GAG-AG	
Intergenic sequence upstream of the Phatr3_J40433 gene	For_J40433	AGG-GAC-TTC-TGT-TAC-ATG-CA	54°C
	Rev_J40433	GGT-TGA-TTT-TCA-AAC-GAT-TG	
Intergenic sequence upstream of the Phatr3_J35771 gene	For_J35771	GGT-GAA-TGC-CCT-CGA-GGG-TG	Ramp from 48°C to 52°C
	Rev_J35771	GAG-TAT-GAC-AAA-CCA-ATA-GG	
Intergenic sequence upstream of the Phatr3_J35102 gene	For_J35102	TAA-TTA-TCA-TTG-TAT-GTT-CG	50°C
	Rev_J35102	GGT-AGA-ACC-AAT-TGG-GTC-GT	
Intergenic sequence upstream of the Phatr3_J8537 gene	For_J8537	GAC-TTC-CGC-AAA-TCC-CCG-AG	62°C
	Rev_J8537	GGC-TGC-CGT-TTT-GAA-GCA-AG	
Intergenic sequence upstream of the Phatr3_J36570 gene	For_J36570	ACG-TAC-GAC-ATT-TTG-GAG-AC	58°C
	Rev_J36570	CTT-ACT-GCT-GAG-GGA-TGA-TA	
Intergenic sequence upstream of the Phatr3_J50252 gene	For_J50252	AAA-TTG-TAT-ACT-CCA-GCA-GT	54°C
	Rev_J50252	GAT-TAC-TGT-AAA-GGG-CAA-GG	
Intergenic sequence upstream of the Phatr3_J49693 gene	For_J49693	CTG-TTG-TAC-ACA-TGA-AAC-CC	58°C
	Rev_J49693	GGG-TAT-TGC-AAG-TCA-GAC-AA	
Intergenic sequence upstream of the Phatr3_J34085 gene	For_J34085	AAT-CAT-AGG-CAA-TGT-TTG-GTA-C	52°C
	Rev_J34085	ATT-TGC-TTG-CTG-TTG-CTG-TTG-G	
Intergenic sequence upstream of the Phatr3_J40539 gene	For_J40539	GGT-TTT-TTG-TGC-TGG-CTT-CC	52°C
	Rev_J40539	GCT-GAA-AAA-ACT-GAA-GTA-TA	
Intergenic sequence upstream of the Phatr3_J46933 gene	For_J46933	GTC-GAC-CAC-GAA-CTG-GTG-AT	56°C
	Rev_J46933	GGT-GAT-TGC-AAA-GGA-TGT-AT	
Intergenic sequence upstream of the Phatr3_J36444 gene	For_J36444	CAA-TAC-CGT-TTC-CTC-ATG-GC	60°C
	Rev_J36444	GGT-AGT-TCC-GGC-GTC-ACT-AT	
Intergenic sequence upstream of the Phatr3_J48164 gene	For_J48164	GTT-GAA-ACT-ACA-TTG-TTG-TC	54°C
	Rev_J48164	ATT-CTC-TAG-GCT-TGT-TTC-TA	
Intergenic sequence upstream of the Phatr3_EG01906 gene	For_EG01906	CTC-TAT-GTG-GTC-AGT-ACT-GGT-C	48°C
	Rev_EG01906	CCC-TAC-ACT-GTA-AAT-TGT-GTT-G	
Intergenic sequence upstream of the Phatr3_EG02422 gene	For_EG02422	TTA-CCA-CCA-AGC-GCT-CTT-CT	54°C
	Rev_EG02422	GTT-CGT-TCT-GTT-TTT-TCG-AT	

Supplementary Table 3. List of primers used in this study to amplify the putative promoter region sequences and the NR promoter (reference promoter in this study), in order to clone them into the pPtPUc3_SLIC-eGFP-NRt vector.

Target sequence	Corresponding Gene ID	Primer's name	5'-3' nucleotide sequences	Hybridization temperature
P_{39391}	Phatr3_J39391	39391_14-LIC_For	TAG-TTG-GAA-TGG-TAC-AAT-GAT-AGT-TGG-TTA-GAT-TGA-CGA-G	59°C
		39391_14-LIC_Rev	TTA-TGG-AGT-TGG-TAC-TGG-AAT-TGG-ATT-AGA-TCT-GGT-GTT-G	
P_{SCF34}	Phatr3_J33266	33266_4-SLIC_For	TAG-TTG-GAA-TGG-TAC-TTT-GAT-TTG-GTT-CGT-CGT-CAC-GAG-T	61°C
		33266_4-SLIC_Rev	TTA-TGG-AGT-TGG-TAC-GGT-TCG-GAA-AAG-TCT-ACC-AGG-TTG-A	
P_{SSR}	Phatr3_J37038	37038_3-SLIC_For	TAG-TTG-GAA-TGG-TAC-TGT-CGC-TAC-TTC-ACC-ACA-GCT-TTA-T	61°C
		37038_3-SLIC_Rev	TTA-TGG-AGT-TGG-TAC-GAT-TCA-GCC-TTG-AAT-CAC-GGT-AGA-G	
P_{46468}	Phatr3_J46468	46468_8-SLIC_For	TAG-TTG-GAA-TGG-TAC-CTG-AAT-AGA-AAT-ATT-TCC-TCC-GCT-G	57°C
		46468_8-SLIC_Rev	TTA-TGG-AGT-TGG-TAC-GGA-GGC-TTT-TGA-AGT-TTT-TTA-TTT-T	
P_{TC}	Phatr3_Jdraft1668	D1668_2-LIC_For	TAG-TTG-GAA-TGG-TAC-CAT-TGT-CCT-ACA-TTG-ATG-ATC-TGA-C	58°C
		D1668_2-LIC_Rev	TTA-TGG-AGT-TGG-TAC-AGC-AAT-GTA-AGT-AAT-TTG-ATA-AAT-AAA-GG	
P_{40651}	Phatr3_J40651	40651_16-SLIC_For	TAG-TTG-GAA-TGG-TAC-ACT-TGC-GTT-CAT-CTC-AAG-TCG-ACT-A	60°C
		40651_16-SLIC_Rev	TTA-TGG-AGT-TGG-TAC-AGT-GAT-ACA-ACA-AAA-TAG-CAG-GTT-CTT-G	
P_{MI}	Phatr3_J42538	42538_9-SLIC_For	TAG-TTG-GAA-TGG-TAC-GAA-GAT-GAG-CAG-TTT-CCG-CAT-CAC-C	63°C
		42538_9-SLIC_Rev	TTA-TGG-AGT-TGG-TAC-GGT-GGT-TGA-GAT-CCG-GGA-TCG-ATG-A	
P_{DUF11}	Phatr3_J48356	48356_16-SLIC_For	TAG-TTG-GAA-TGG-TAC-CCT-TTA-GGA-GAA-CAA-GCT-GCG-GAA-G	61°C
		48356_16-SLIC_Rev	TTA-TGG-AGT-TGG-TAC-CTT-CGC-TGC-AGC-CTT-TTG-GTA-AAA-T	
P_{33783}	Phatr3_J33783	33783_15-SLIC_For	TAG-TTG-GAA-TGG-TAC-GAC-CGT-GAC-GAC-GGA-TTG-TTT-TGG	62°C
		33783_15-SLIC_Rev	TTA-TGG-AGT-TGG-TAC-TGT-TGT-CGA-ATA-ATG-ATG-GCA-CCG-G	
P_{41599}	Phatr3_J41599	41599_15-SLIC_For	TAG-TTG-GAA-TGG-TAC-GAT-GAG-TAC-AGA-TCA-GGC-ACA-ATC-A	61°C
		41599_15-SLIC_Rev	TTA-TGG-AGT-TGG-TAC-GAT-GTT-ACT-AGA-AAC-GAA-TCA-CTC-GG	
P_{50361}	Phatr3_J50361	50361_20-SLIC_For	TAG-TTG-GAA-TGG-TAC-CGT-CCT-TAC-GGC-CCC-TTT-GTG-GTA	61°C
		50361_20-SLIC_Rev	TTA-TGG-AGT-TGG-TAC-GTC-AAT-AAA-CGA-TCT-CCT-GAA-TCT-GAC-AA	
$P_{DUF2711}$	Phatr3_J43621	43621_4-SLIC_For	TAG-TTG-GAA-TGG-TAC-ACC-ACG-TCA-ATT-GCG-TAA-TTC-GTC-A	61°C

		43621_4-SLIC_Rev	TTA-TGG-AGT-TGG-TAC-GGT-GCG-GAA-ATC-AAG-TTT-TCG-AGG-T	
P_{AP1}	Phatr3_J34976	34976_18-SLIC_For	TAG-TTG-GAA-TGG-TAC-TGA-CCA-AGG-TAC-GAA-GCT-CTC-CGT-A	62°C
		34976_18-SLIC_Rev	TTA-TGG-AGT-TGG-TAC-TGG-TTT-GTC-ATC-AAC-TGG-CTT-GGT-TG	
P_{RHFA}	Phatr3_EG02507	E2507_1-SLIC_For	TAG-TTG-GAA-TGG-TAC-TTC-CGA-TGC-GTG-TGT-TTG-TAA-AGG-C	61°C
		E2507_1-SLIC_Rev	TTA-TGG-AGT-TGG-TAC-TTT-GTG-GTT-CGA-CGC-GAA-TCA-TAG-A	
P_{PMSR}	Phatr3_J8683	8683_3-SLIC_For	TAG-TTG-GAA-TGG-TAC-GTT-GGG-AGA-ACC-ACT-ATC-GAG-GAA-A	61°C
		8683_3-SLIC_Rev	TTA-TGG-AGT-TGG-TAC-GAG-ATT-TGT-GTG-GTC-ATA-CCT-GGT-TT	
P_{PCNTH}	Phatr3_Jdraft1443	D1443_6-SLIC_For	TAG-TTG-GAA-TGG-TAC-TCG-GGT-TGG-AGT-ACT-GCT-AGG-AGC-A	63°C
		D1443_6-SLIC_Rev	TTA-TGG-AGT-TGG-TAC-AGT-CGT-GGA-ATG-GAT-GAG-AGT-TGT-TTC-C	
P_{SCF34}	Phatr3_J40433	40433_20-SLIC_For	TAG-TTG-GAA-TGG-TAC-AGG-GAC-TTC-TGT-TAC-ATG-CAT-ATT-TCT-TC	60°C
		40433_20-SLIC_Rev	TTA-TGG-AGT-TGG-TAC-GGT-TGA-TTT-TCA-AAC-GAT-TGC-TCG-A	
P_{35102}	Phatr3_J35102	35102_3-SLIC_For	TAG-TTG-GAA-TGG-TAC-TAA-TTA-TCA-TTG-TAT-GTT-CGT-TCT-CTA-GGA-AT	59°C
		35102_3-SLIC_Rev	TTA-TGG-AGT-TGG-TAC-GGT-AGA-ACC-AAT-TGG-GTC-GTC-GG	
P_{SST5}	Phatr3_J8537	8537_14-SLIC_For	TAG-TTG-GAA-TGG-TAC-GAC-TTC-CGC-AAA-TCC-CCG-AGG-TAT-T	62°C
		8537_14-SLIC_Rev	TTA-TGG-AGT-TGG-TAC-GGC-TGC-CGT-TTT-GAA-GCA-AGA-TTG-A	
P_{SH}	Phatr3_J36570	36570_24-SLIC_For	TAG-TTG-GAA-TGG-TAC-ACG-TAC-GAC-ATT-TTG-GAG-ACG-AGC	62°C
		36570_24-SLIC_Rev	TTA-TGG-AGT-TGG-TAC-CTT-ACT-GCT-GAG-GGA-TGA-TAC-TAA-CAA-TAC-TG	
P_{GSTMu3}	Phatr3_J50252	50252_20-SLIC_For	TAG-TTG-GAA-TGG-TAC-AAA-TTG-TAT-ACT-CCA-GCA-GTC-GTT-T	59°C
		50252_20-SLIC_Rev	TTA-TGG-AGT-TGG-TAC-CAT-TAC-TGT-AAA-GGG-CAA-GGA-ATT-C	
P_{VOC}	Phatr3_J34085	34085_11-SLIC_For	TAG-TTG-GAA-TGG-TAC-AAT-CAT-AGG-CAA-TGT-TTG-GTA-CAC-AAT	59°C
		34085_11-SLIC_Rev	TTA-TGG-AGT-TGG-TAC-ATT-TGC-TTG-CTG-TTG-CTG-TTG-GTT-TAA	
P_{40539}	Phatr3_J40539	40539_19-SLIC_For	TAG-TTG-GAA-TGG-TAC-GGT-TTT-TTG-TGC-TGG-CTT-CCA-GTA-G	61°C
		40539_19-SLIC_Rev	TTA-TGG-AGT-TGG-TAC-GCT-GAA-AAA-ACT-GAA-GTA-TAA-GGA-AAA-AGC-AC	
P_{46933}	Phatr3_J46933	46933_22-SLIC_For	TAG-TTG-GAA-TGG-TAC-GTC-GAC-CAC-GAA-CTG-GTG-ATG-CCC	63°C
		46933_22-SLIC_Rev	TTA-TGG-AGT-TGG-TAC-GGT-GAT-TGC-AAA-GGA-TGT-ATG-TGT-TGA-CTT-TG	
P_{36444}	Phatr3_J36444	36444_6-SLIC_For	TAG-TTG-GAA-TGG-TAC-CAA-TAC-CGT-TTC-CTC-ATG-GCT-GGT-C	63°C
		36444_6-SLIC_Rev	TTA-TGG-AGT-TGG-TAC-GGT-AGT-TCC-GGC-GTC-ACT-ATG-TGC	
P_{HDC}	Phatr3_J48164	48164_2-SLIC_For	TAG-TTG-GAA-TGG-TAC-GTT-GAA-ACT-ACA-TTG-TTG-TCG-GAT-G	59°C

		48164_2-SLIC_Rev	TTA-TGG-AGT-TGG-TAC-ATT-CTC-TAG-GCT-TGT-TTC-TAC-ACA-AC	
P_{E1906}	Phatr3_EG01906	E1906_1-SLIC_For	TAG-TTG-GAA-TGG-TAC-GTG-GTC-AGT-ACT-GGT-CTG-ATA-TAG-ATT	60°C
		E1906_1-SLIC_Rev	TTA-TGG-AGT-TGG-TAC-CCC-TAC-ACT-GTA-ATT-GTG-TTG-TCG-T	
$P_{PCNTH-2}$	Phatr3_EG02422	E2422_6-SLIC_For	TAG-TTG-GAA-TGG-TAC-TTA-CCA-CCA-AGC-GCT-CTT-CTA-TCT-A	60°C
		E2422_6-SLIC_Rev	TTA-TGG-AGT-TGG-TAC-GTT-CGT-TCT-GTT-TTT-TCG-ATA-GAG-TTG-C	

**Target sequence**	**Primer's name**	**5'-3' nucleotide sequences**	**Hybridization temperature**
P_{NR}	NR_Prom-SLIC_For	TAG-TTG-GAA-TGG-TAC-CAT-ATG-CGG-AAG-TGA-CTG-TAA-ACG-AGA-AG	62°C
	NR_Prom-SLIC_Rev	TTA-TGG-AGT-TGG-TAC-CGT-TCG-CAC-AAG-TGG-TGA-CTT-TGA-A	
